# Stem Cell Therapy for Traumatic Brain Injury: Translational Mechanisms, Biomarkers, and Clinical Trials

**DOI:** 10.1007/s12015-026-11175-9

**Published:** 2026-06-18

**Authors:** William T. Creel, Sukhpreet Randhawa, Richard E. Hartman

**Affiliations:** Department of Psychology, Loma Linda University, Loma Linda, CA 92354 USA

**Keywords:** Traumatic Brain Injury, Stem Cells, Biomarkers, Neurorestoration, Clinical Trials

## Abstract

Traumatic brain injury (TBI) is a leading cause of persistent cognitive, motor, and neuropsychiatric impairment, arising from both the initial mechanical insult and a prolonged cascade of secondary injury processes. While primary injury reflects structural damage, secondary mechanisms, including neuroinflammation, oxidative stress, apoptosis, and blood-brain barrier disruption, evolve over time and critically influence long-term outcomes. Despite extensive investigation, therapies targeting isolated components of secondary injury have demonstrated limited clinical success, highlighting the need for approaches that support coordinated neural repair. Stem cell-based therapies have emerged as a promising neurorestorative strategy. Rather than replacing lost neurons directly, transplanted cells primarily act through paracrine signaling, immunomodulation, neurotrophic support, and preservation of vulnerable neural networks. However, clinical translation has been constrained by heterogeneity in cell sources, delivery routes, treatment timing, and outcome measures, limiting mechanistic interpretability across trials. This review presents a mechanism-aligned translational framework linking injury stage, biological targets, route of administration, and clinically meaningful outcomes across stages of TBI. We integrate current knowledge of post-injury repair mechanisms with evidence from recent human clinical trials of stem cell therapies. Particular emphasis is placed on safety, feasibility, and emerging signals across structural and functional endpoints, as well as how injury stage, biological targets, delivery strategies, and meaningful endpoints interact to shape treatment responsiveness and inform biomarker-guided clinical trial design.

## Background

### Traumatic Brain Injury as a Disorder of Progressive Neural Damage

Traumatic brain injury (TBI) refers to a broad class of injuries resulting from external mechanical forces that disrupt normal brain structure and function. These injuries arise from diverse mechanisms, including blunt impact, rapid acceleration-deceleration, rotational forces, and penetrating trauma, and occur across a wide range of contexts with leading causes including motor vehicle collisions, falls, sports-related injuries, and assaults [1]. Collectively, TBI represents a major public health burden and a leading cause of injury-related morbidity and mortality, with substantial individual, societal, and economic consequences [2].

Importantly, TBI is not a singular pathological event, but a heterogeneous disorder characterized by variability in injury mechanisms, anatomical involvement, and clinical presentation. Although the initial mechanical insult produces immediate structural disruption, the neurological consequences of TBI are not determined solely at the moment of impact. Rather, brain injury unfolds dynamically over time through a series of delayed biological processes that extend well beyond the acute phase. These evolving secondary injury mechanisms critically influence neuronal survival, network integrity, and long-term functional outcome, reframing TBI as a disorder of progressive neural damage rather than a static traumatic event. Since the dominant biology shifts across injury stages, therapeutic effects and the endpoints used to detect them must be interpreted in relation to injury timing, mechanistic target engagement, and the route of administration.

### Effects of Traumatic Brain Injury

TBI is commonly conceptualized as a two-stage process involving immediate primary tissue disruption and secondary injury mechanisms that unfold over time and amplify damage beyond the initial lesion [3, 4]. Although decades of research have identified numerous therapeutic targets within this cascade, translation into consistently effective clinical interventions has remained limited, reflecting the multifactorial nature of secondary injury and the difficulty of modifying complex, time-dependent biological processes with single-mechanism treatments [4]. Secondary injury emerges within minutes and can evolve over hours to days, involving molecular, inflammatory, and neurochemical alterations that drive continued neuronal loss and network disruption [4].

### Excitotoxicity, Trophic Failure, and Cell Degeneration

A key early feature of secondary injury is neuronal membrane depolarization and excessive release of excitatory neurotransmitters (e.g., glutamate and aspartate), producing calcium influx and downstream activation of destructive enzymatic pathways [5]. These processes contribute to mitochondrial dysfunction, oxidative stress, protease and caspase activation, and ultimately apoptotic and necrotic cell death. The resulting cellular degeneration contributes to progressive neuronal loss and provides a mechanistic basis for many of the cognitive, behavioral, and neurological deficits observed following TBI.

Beyond direct cell loss, secondary injury can also disrupt endogenous repair signaling, including reduced neurotrophic support and impaired growth factor-mediated recovery processes. This “trophic failure” can limit neuronal survival, axonal integrity, and synaptic remodeling, helping explain why interventions that restore pro-survival and pro-plasticity signaling may be beneficial.

### Immune Dysregulation, Neuroinflammation, and Blood-Brain Barrier Disruption

Following this early excitotoxic cascade, secondary injury is further propagated by neuroinflammatory signaling and disruption of the blood-brain barrier (BBB), which together contribute to worsening tissue vulnerability and impaired cerebral homeostasis [4]. BBB compromise increases vascular permeability and promotes vasogenic edema, which can elevate intracranial pressure and exacerbate ischemic and metabolic stress within already vulnerable neural tissue [4, 6]. These vascular and inflammatory processes are therefore central contributors to the progression of secondary injury and represent key targets for neuroprotective and neurorestorative interventions.

Beyond the acute phase, secondary injury processes may contribute to long-term neurobiological change, including premature brain aging and neurodegenerative-like pathology. In juvenile animal models, early vascular injury has been shown to produce persistent alterations in BBB phenotype, disrupted protein trafficking, and cognitive impairment [6]. Notably, BBB disruption in the developing brain has been associated with decreased P-glycoprotein expression in cortical blood vessels and increased beta-amyloid accumulation over time, suggesting a pathway through which early injury may increase vulnerability to progressive cognitive decline [6]. These findings underscore the importance of therapeutic strategies that preserve BBB integrity and limit chronic inflammatory and degenerative signaling following TBI.

### Network Disruption

Conventional neuroimaging with computed tomography (CT) and standard magnetic resonance imaging (MRI) may fail to detect acute abnormalities in uncomplicated concussion; however, advanced MRI approaches have demonstrated evidence of mild axonal injury in some cases, supporting the presence of microstructural disruption even in the absence of overt imaging findings [7]. This dissociation between “normal” standard imaging and persistent symptoms underscores that network-level disruption can occur even when macroscopic structural lesions are not apparent.

In addition to diffuse secondary cascades, TBI encompasses distinct structural injury subtypes that contribute to heterogeneity in clinical presentation and outcome. Extra-axial hematomas, including epidural and subdural hematomas, result from vascular rupture and intracranial bleeding and may produce focal neurological deficits such as speech disturbance, hemiparesis, seizures, confusion, and altered mental status depending on lesion location and mass effect [4]. Contusions may be accompanied by subarachnoid hemorrhage due to capillary tearing during coup-contrecoup injury, with potential downstream effects including reduced cerebral blood flow and increased risk of secondary ischemic complications; cognitive and emotional sequelae may include headaches, depression, memory deficits, personality change, and emotional lability [8]. Although these injuries can be focal, their functional consequences often extend beyond the lesion site through disruption of distributed networks supporting cognition, affect regulation, and motor control.

Diffuse axonal injury (DAI) is classically associated with rotational acceleration-deceleration forces and involves widespread axonal shearing that disrupts white matter connectivity [9]. More severe axonal disruption can produce prolonged loss of consciousness, whereas milder forms may result in persistent functional impairment, including difficulty with social reintegration, reduced productivity, and diminished quality of life [4, 10]. Collectively, these patterns highlight that long-term disability following TBI reflects both focal structural lesions and diffuse network-level disruption, reinforcing the need for interventions that support coordinated repair across multiple biological domains that preserve network stability.

In addition to cognitive and motor impairment, TBI is frequently associated with persistent neuropsychiatric sequelae, including depression and affective dysregulation, which may occur even following mild injury. In animal models, single and repetitive mild concussive injuries have been shown to produce depression-like behavioral changes that persist for weeks to months, potentially reflecting downstream effects of neuroinflammation, oxidative stress, apoptosis, and cytokine dysregulation within secondary injury cascades [11]. These findings underscore that long-term disability after TBI reflects both structural damage and evolving network-level dysfunction. Accordingly, outcome domains that capture network integrity and recovery trajectories (e.g., white matter microstructure, connectivity, and cognitive/behavioral function) may be especially relevant when evaluating restorative interventions aimed at network preservation.

### Effects of Multiple TBI’s

Repeated mild traumatic brain injury (mTBI) is a major clinical concern due to its cumulative effects on tissue integrity and functional recovery, particularly in high-risk populations such as military personnel and contact-sport athletes [12]. Experimental evidence suggests that the brain may enter a time-limited period of heightened vulnerability following an initial mTBI, during which a subsequent injury can produce disproportionate neuropathological consequences [13, 14]. Using controlled cortical impact lesions delivered to opposite hemispheres, Donovan and colleagues [13] found that a second contralateral injury administered days after the first altered the spatial and pathological profile of injury expression, consistent with a post-injury vulnerability window. Notably, repeated mTBI was associated with increased blood deposition and colocalization with activated microglia, suggesting that vascular disruption and neuroinflammatory amplification contribute to tissue damage extending beyond the focal injury site [13].

Complementary findings have been reported in other repetitive injury paradigms, in which two mild TBIs delivered within short inter-injury intervals produced cumulative harm, including increased lesion and hemorrhagic burden and persistent behavioral impairment [14]. Together, these studies support the view that repetitive mTBI is not simply additive but may reflect a temporally dependent amplification of secondary injury mechanisms, with implications for both clinical management and therapeutic timing [13, 14].

The cumulative consequences of repeated mTBI likely reflect overlapping cell death and axonal injury pathways that unfold across multiple temporal phases. Because these mechanisms are interconnected and redundant, inhibiting a single pathway has generally shown limited benefit, contributing to repeated failures in clinical neuroprotection trials [15–17]. These findings further underscore the need for therapies capable of supporting coordinated repair across inflammatory, vascular, and network-level domains. From a translational perspective, the heterogeneity of injury biology implies that therapeutic success depends less on a single cell product and more on aligning mechanism, injury stage, and measurable biomarkers of response. This review, therefore, approaches stem cell therapy not as a discrete intervention, but as a framework for examining mechanism-guided neurorestorative strategies.

### Current Treatments for TBI

Current clinical management of TBI is multifaceted and depends on injury severity, lesion type, and evolving secondary pathophysiology. In the acute setting, treatment is primarily aimed at preventing secondary injury by optimizing cerebral perfusion and oxygenation, controlling intracranial pressure, and surgically addressing mass lesions such as hematomas or contusions when indicated [4]. Prophylactic antiepileptic therapy is also commonly used to reduce the risk of early post-traumatic seizures and is typically discontinued after the first week post-injury [18].

Beyond acute stabilization, rehabilitation and adjunctive interventions play an important role in functional recovery, with growing interest in approaches that modulate neural excitability and plasticity. Hyperbaric oxygen therapy has been investigated for its potential to support metabolic recovery and reduce inflammatory and cell death pathways, although findings remain mixed and efficacy has not been conclusively established across injury severities and outcome measures [19]. Noninvasive brain stimulation techniques, including transcranial magnetic stimulation (TMS) and transcranial direct current stimulation (tDCS), have also been explored as promising tools to support recovery by enhancing or suppressing cortical excitability within targeted networks [20, 21]. Early studies suggest these interventions may improve select cognitive and neuropsychiatric outcomes in some patients, though optimal stimulation parameters, patient selection, and durability of effects remain active areas of investigation.

Despite these advances, many interventions primarily focus on limiting secondary injury or facilitating compensatory recovery, although few therapies directly support coordinated biological repair across inflammatory, vascular, and synaptic domains. This ongoing gap motivates investigation of neurorestorative strategies aimed at supporting coordinated repair processes beyond acute neuroprotection [22].

**Fig. 1:**
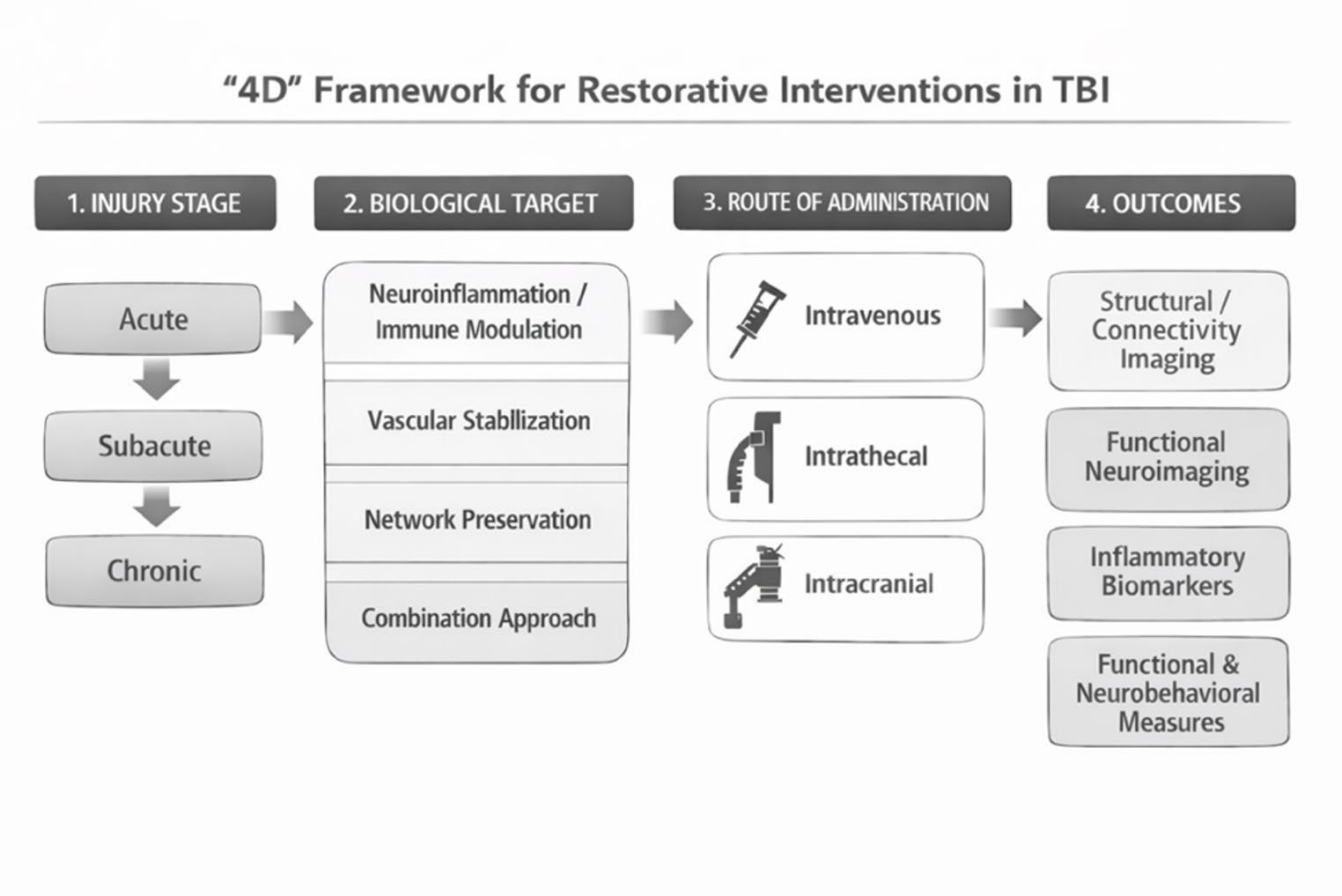
“4D” Framework for Restorative Interventions in TBI. A translational framework for evaluating restorative interventions in TBI, organized around four interdependent dimensions: injury stage, biological target, route of administration, and outcome selection

A translational framework for studying restorative interventions in TBI can be conceptualized around four interdependent dimensions (see Fig. 1): (1) injury stage, defining whether the patient is in the acute, subacute, or chronic phase; (2) biological target, specifying which mechanisms are most actionable at that stage (e.g., neuroinflammation/immune modulation, vascular stabilization, network preservation, or a combination); (3) route of administration, selected to maximize measurable target engagement and trial interpretability; and (4) outcomes, defined a priori to capture both mechanistic engagement and clinically meaningful benefit, including structural/connectivity measures (e.g., diffusion tensor imaging), functional neuroimaging, inflammatory biomarkers, and standardized functional or neurobehavioral measures.

### Stem Cells Used in TBI Research

In recent years, stem cell-based therapies have gained increasing attention as candidate neurorestorative interventions for TBI, with preclinical and early clinical studies reporting improvements in functional recovery across cognitive, motor, and neurobehavioral domains [23]. A broad range of stem and progenitor cell populations have been evaluated in TBI models, including mesenchymal stem/stromal cells (MSCs), multipotent adult progenitor cells (MAPCs), endothelial progenitor cells (EPCs), neural stem cells (NSCs), and emerging research on induced pluripotent stem cell (iPSC)-derived neural cells [23, 24]. Although early efforts emphasized cellular replacement, accumulating evidence suggests that many observed benefits are mediated predominantly through paracrine signaling, immunomodulation, neurotrophic support, and preservation of vulnerable neural and vascular networks rather than direct engraftment and neuronal integration [23, 25, 26].

### Multipotent Adult Progenitor Cells (MAPCs)

MAPCs are self-renewing cells originally isolated from adult bone marrow and other tissues, with the capacity to differentiate across multiple lineages under specific conditions [24]. In experimental TBI models, intravenous MAPC administration has been associated with improvements in spatial learning and memory, as well as motor-related outcomes at extended post-injury time points [27]. Mechanistically, MAPCs appear to exert particularly strong effects through systemic immunomodulation, driven in part by splenocyte-mediated signaling that shifts the post-injury immune milieu toward an anti-inflammatory phenotype [28]. This immune reprogramming has been linked to increases in anti-inflammatory cytokines (e.g., IL-4, IL-10), expansion of regulatory immune activity, and downstream changes of neuroinflammatory responses toward more reparative profiles, including increased alternatively activated microglia/macrophages (M2-like) and reduced pro-inflammatory activation [27, 28].

In parallel, MAPCs have demonstrated robust BBB and microvascular stabilization during the acute post-injury period, with evidence that BBB preservation may be mediated through MAPC-splenocyte interactions that modulate splenic efflux and attenuate inflammatory injury to the cerebral microvasculature [28]. Importantly, MAPC efficacy is highly time-sensitive, with the therapeutic window occurring within the first 24 h after injury. In timing optimization work, administration at 2 and 24 h post-injury produced superior BBB preservation, cognitive improvement, and attenuation of activated microglia/macrophages compared to later dosing schedules, with efficacy declining substantially as treatment is delayed beyond this early window [29]. Collectively, these findings position MAPCs as a promising acute, immune-dominant intervention targeting diffuse secondary injury cascades, supported by early-phase translational development and preclinical timing characterization [29].

### Mesenchymal Stem/Stromal Cells (MSCs)

Mesenchymal stem/stromal cells (MSCs) are heterogeneous, multipotent adult progenitor cells that can be derived from multiple tissue sources, including bone marrow, adipose tissue, perivascular niches, and umbilical cord/cord blood [23, 25]. MSCs are widely studied in neurotrauma due to their relative accessibility, scalability, and immunomodulatory profile, as well as their capacity to secrete trophic factors that support tissue repair and synaptic plasticity [23]. Functionally, MSCs may mitigate secondary injury after TBI primarily through immunomodulation and neurovascular/trophic support, mechanisms that align with key inflammatory and repair processes driving post-injury pathology [23, 30].

MSC-mediated immunomodulation represents a primary mechanism of therapeutic benefit in TBI. MSCs reduce pro-inflammatory cytokines (IL-1β, IL-6, TNF-α) and increase anti-inflammatory cytokines (IL-10, TGF-β1) through secretion of tumor necrosis factor-α stimulated gene/protein 6 (TSG-6), which suppresses nuclear factor-kB (NF-kB) signaling and attenuates secondary neuroinflammatory cascades [31]. In parallel, MSC effects on inflammatory pathways appear context-dependent, with evidence that intrathecal MSC implantation can engage NF-kB-linked cytokine signaling (including IL-6) in a manner associated with enhanced neuroprotection [30]. Collectively, this immunomodulatory profile is associated with reduced neuroinflammation and decreased microglial activation, neutrophil infiltration, and macrophage recruitment within injured tissue.

Beyond immune effects, MSCs support BBB integrity through reduced inflammatory signaling and preservation of tight junction proteins, while also releasing a broad spectrum of neurotrophic and angiogenic factors including brain-derived neurotrophic factor (BDNF), vascular endothelial growth factor (VEGF), nerve growth factor (NGF) [32, 33]. These factors are delivered via paracrine signaling and extracellular vesicles, enabling MSCs to promote neurogenesis, angiogenesis, and stabilization of vulnerable neural networks across multiple biological domains.

In preclinical TBI studies, MSCs have been shown to migrate preferentially toward injured brain regions and to contribute to functional improvement, with some reports describing differentiation into neural lineage markers, including astrocytic and neuronal phenotypes [34]. MSC therapy has also been associated with reduced neuroinflammation and decreased markers of cellular injury, including reductions in microglial activation, neutrophil infiltration, macrophage recruitment, and pro-inflammatory signaling within injured tissue [31]. Across multiple experimental paradigms, MSC administration has been reported to reduce apoptosis, decrease lesion burden, and improve behavioral recovery [35, 36]. Collectively, these findings have positioned MSCs among the most extensively investigated cell types for modulating secondary injury mechanisms and promoting functional recovery after TBI.

These findings are supported across multiple independent meta-analyses of preclinical cell therapy studies in TBI. One study confirmed significant MSC effects on sensorimotor and neurological motor function across 28 studies [37], while a broader analysis, extending to multiple progenitor cell types across 38 studies, found large treatment effects on lesion volume and neurological severity [38]. The most comprehensive meta-analysis to date confirmed improvements across sensorimotor, cognitive, and anatomical outcomes, with greater effect sizes observed for first-week administration and direct intralesional delivery [39].

### Bone Marrow-Derived MSCs (BM-MSCs)

Bone marrow-derived MSCs (BM-MSCs) have been widely studied as an alternative to embryonic stem cell approaches and have been shown to release neurotrophic and angiogenic factors that support repair in injured neural tissue [32, 33]. In human and experimental transplantation contexts, BM-MSCs have been associated with increased expression of growth factors and cytokines, including BDNF, VEGF, NGF, and hepatocyte growth factor (HGF), which may contribute to reduced apoptosis and enhanced angiogenesis [32, 33]. Importantly, BM-MSC efficacy at chronic timepoints (~ 2 months post-injury) appears critically dependent on delivery route and injury severity [40, 41]. At this stage, intralesional transplantation has been shown to promote neurogenesis and functional recovery [40], whereas delayed intravenous delivery failed to improve neurological, neurobehavioral, or histological outcomes [42, 43]. Experimental TBI studies further suggest that BM-MSC injection may produce functional benefits in adult rats with moderate brain damage, but not in animals with severe injury [41]. Together, these findings indicate that route selection should be considered in relation to both therapeutic timing and injury severity, and that intravenous delivery should not be assumed to be effective in chronic TBI without evidence of adequate tissue targeting.

### Umbilical Cord-Derived MSCs (UC-MSCs)

Umbilical cord-derived MSCs (UC-MSCs) can be obtained from umbilical cord tissue or cord blood and offer practical advantages, including noninvasive collection, high proliferative capacity, and relatively low immunogenicity [44, 45]. UC-MSCs secrete multiple neurotrophic and immunoregulatory factors that may support neuronal survival, modulate the local immune response, and promote recovery within injured brain networks [46]. These properties have contributed to growing interest in UC-MSCs as a scalable and potentially clinically translatable cell source.

### Adipose-Derived MSCs (AD-MSCs)

Adipose-derived MSCs (AD-MSCs) represent another highly accessible MSC population, obtained in relatively large quantities and capable of differentiation toward neural and vascular-associated phenotypes under appropriate conditions [25, 47]. In experimental TBI models, AD-MSCs have been associated with improved motor outcomes, reduced neuronal cell death, and modulation of inflammatory responses [48]. AD-MSCs also secrete cytokines and growth factors, including BDNF, which may contribute to attenuation of neuroinflammatory signaling after injury [49].

### Induced Pluripotent Stem Cells (iPSCs)

iPSCs are derived from adult somatic cells and were developed in part to address ethical and logistical constraints associated with embryonic stem cells [25]. iPSC-derived neural cells have shown promising results in experimental TBI paradigms, including survival following transplantation, migration toward injured tissue, and functional improvement in motor outcomes [50, 51]. However, iPSC-based approaches remain constrained by manufacturing complexity, cost, and translational considerations, including safety and consistency across differentiated cell products [25]. These challenges highlight the importance of continued optimization of differentiation protocols and rigorous evaluation of long-term safety profiles.

### Endothelial Progenitor Cells (EPCs)

EPCs contribute to vascular repair through the regeneration of endothelial lining and support of angiogenic processes. EPCs originate largely from bone marrow and can differentiate into vascular endothelial cells, with mobilization and recruitment influenced by chemokines and adhesion signaling following injury [52, 53]. Given the central role of BBB disruption and microvascular dysfunction in secondary injury, EPCs have received increasing attention as a vascular-targeted regenerative strategy.

Mechanistically, preclinical work suggests EPCs may support BBB restoration not only through endothelial integration, but also through strengthening barrier integrity via tight junction recovery (e.g., ZO-1 and claudin-5) and vascular stabilization signaling, including shifts toward a higher Ang1:Ang2 ratio, changes consistent with improved endothelial cohesion and vessel maturation [54]. In parallel, EPCs exhibit a strongly angiogenic profile, with signaling linked to VEGF and Ang-1 pathways that support endothelial sprouting, neovascularization, and stabilization of vulnerable microvascular networks after injury [54].

Circulating EPC levels have been proposed as a marker of endothelial injury and BBB disruption after TBI [55]. In clinical observational work, EPC counts have been reported to increase in the early post-injury period, with lower EPC levels associated with worse outcomes, suggesting potential prognostic relevance [56]. EPC transplantation approaches have also incorporated exogenous factors (e.g., progesterone, erythropoietin) to enhance migration and vascular integration, supporting the broader concept that vascular repair may represent a key pathway toward improved neurological recovery [57].

### Neural Stem Cells (NSCs)

Neural stem cells (NSCs) are multipotent stem cells capable of self-renewal and differentiation into neurons, astrocytes, and oligodendrocytes [52]. NSCs can be derived from embryonic or adult neural tissue and can also be generated from embryonic stem cell platforms [58]. NSCs are of particular interest in neurotrauma due to their lineage proximity to CNS cell types and their potential to support both replacement and trophic mechanisms depending on context.

NSCs exert therapeutic benefits through targeted secretion of neurotrophic factors, particularly glial cell line-derived neurotrophic factor (GDNF) and BDNF. In TBI models, GDNF has been associated with reduced traumatic axonal injury and pathological α-smooth muscle actin (α-SMA) expression, thereby protecting axons from secondary degeneration, whereas BDNF promotes synaptic plasticity and neuronal survival via TrkB receptor signaling [59–62]. Although NSCs do not primarily stabilize the BBB through direct endothelial integration, their neurotrophin-driven axonal preservation and microenvironmental support may secondarily promote barrier integrity by modulating microglial activation and reducing injury propagation, neuroinflammatory signaling, and vascular stressors that contribute to BBB disruption [52, 61].

Transplantation studies have demonstrated that human NSCs can survive for extended periods in rodent TBI models, with reported survival ranging from approximately two to five months in some paradigms [63]. In addition, NSC transplantation has been associated with sustained cognitive improvement in experimental models [52, 63]. NSCs may also be engineered to enhance therapeutic effects; for example, NSCs transfected with neurotrophic genes have been shown to increase pyramidal cell survival in the hippocampus and improve learning and motor outcomes in experimental injury models [59].

Timing appears to be a critical determinant of NSC efficacy. In experimental work, NSC transplantation at approximately 9 days post-injury has been associated with greater improvements in motor and cognitive recovery than later delivery (e.g., 14 days), whereas transplantation at 1-month post-injury has shown minimal benefit in some paradigms [64]. This narrow therapeutic window suggests that NSCs are most effective when targeting focal lesions with preserved perilesional circuits during early post-injury phases when endogenous plasticity mechanisms remain active.

A key translational consideration for NSC-based approaches is safety, including the theoretical risk of uncontrolled proliferation and tumorigenesis associated with highly self-renewing cell populations [65]. Noninvasive monitoring strategies such as MRI-based cell tracking have been developed to assess cell location, migration, and proliferation over time, including approaches using iron-oxide labeling for longitudinal monitoring in vivo [66]. These advances support the feasibility of safety surveillance in experimental transplantation paradigms and may inform future translational development.

Finally, biological moderators of treatment response, including host factors such as sex, have received increasing attention. In neonatal hypoxic-ischemic injury models treated with human female NSCs, Ashwal and colleagues [67] reported no significant sex differences in therapeutic response, supporting the potential for broad applicability across male and female recipients in at least some injury contexts.

Across diverse stem and progenitor cell platforms, preclinical evidence supports the potential for stem cell-based interventions to modulate secondary injury mechanisms, preserve neurovascular integrity, and promote functional recovery after TBI. Differences in cell source, delivery timing, and injury state likely contribute to variability in observed outcomes, emphasizing the importance of mechanistically informed patient selection and biomarker-guided targeting in future translational work [23, 25]. A stem cell mechanistic summary is provided in Table 1.

**Table 1:** Stem cell mechanistic summary in TBI. Summary of the four primary platforms evaluated in TBI research (MSCs, NSCs, EPCs, MAPCs) across immune modulation, BBB stabilization, trophic support, network preservation, timing, lesion context, and evidence level. iPSC-derived neural cells are an emerging platform but remain largely preclinical and are not included due to translational constraints (e.g., manufacturing complexity and safety considerations)

Mechanism	MSCs	NSCs	EPCs	MAPCs
Immune Modulation	Direct: Reduces pro-inflammatory cytokines; increases anti-inflammatory cytokines via TSG-6/NF-κB pathway^30,31^	Indirect: Via microglial modulation; improves local microenvironment^52,61^	Incidental: Primarily vascular; modest paracrine immune effects^53,54^	Direct: Splenocyte-mediated shift to anti-inflammatory phenotype; increases Treg cells; M2 microglia^27,28^
BBB Stabilization	Indirect: Preserves tight junction proteins via immunomodulation and trophic support^31,32,33^	Indirect: Via neurotrophin-mediated axonal preservation and microenvironmental support^52,61^	Direct: Via endothelial integration; restores ZO-1, claudin-5; increases Ang1:Ang2 ratio^54^	Direct: Preservation via splenocyte interaction; stabilizes microvasculature^28^
Trophic Support	Direct (Broad): BDNF, VEGF, NGF, HGF, FGF, IGF-1; supports neurogenesis and angiogenesis^32,33^	Direct (Targeted): GDNF-associated axonal protection; BDNF supports synaptic plasticity via TrkB^59,60,61,62^	Direct (Angiogenic): VEGF, Ang-1; promotes endothelial sprouting and vessel maturation^54^	Indirect (Systemic): Primarily through splenocyte-mediated systemic immunomodulation^27,28^
Network Preservation	Indirect: Supports synaptic maintenance and circuit recovery via neurotrophic support and inflammation reduction^31,32,33,34^	Direct: Promotes circuit repair via neurotrophin secretion and local microenvironment support; may enhance synaptic remodeling and axonal preservation^60,61,62^	Indirect: Primarily neurovascular stabilization; network-level benefits likely secondary to improved perfusion and BBB integrity^52,53,54^	Indirect: Preserves network stability via systemic immunomodulation and reduced secondary injury burden, supporting downstream circuit function^27,28,29^
Optimal Timing	Days 3-60^23,25^	Days 2-10^64^	Unknown^52,53^	Hours 2-24^29^
Lesion Context	Focal or diffuse^23,25^	Focal with preserved penumbra^64,68^	Acute vascular-dominant^54,55^	Diffuse; immune-dominant cascades^27,28^
Evidence Level	Phase 2 RCT; multiple Phase 1; robust preclinical; three independent meta-analyses^37,38,39^	Phase 1–2 preclinical; timing from single study^63,64^	Phase 1 observational/animal; no RCT transplantation data^54,56,57^	Phase 1; rigorous timing optimization^27,28,29^

### Mechanism of Action

A central challenge in translating stem cell therapies to TBI treatment is identifying which patients are most likely to benefit based on the biological state of the injured brain and the therapeutic mechanisms plausibly engaged by the administered cell product. In principle, this requires aligning candidate mechanisms (e.g., immunomodulation, vascular stabilization, trophic support, and network preservation) with biomarkers that indicate the presence of salvageable tissue and an injury milieu receptive to repair and avoiding treatment in patients whose lesion profile suggests limited capacity for benefit [68]. Critically, therapeutic efficacy is not solely a property of the cell product itself, but of its alignment with injury stage and target biology, including the timing and route of administration needed to engage the dominant mechanisms at a given phase of recovery. Although definitive clinical selection algorithms remain under development, emerging preclinical work has begun to address this translational gap. To operationalize this mechanism-aligned approach, Table 2 summarizes candidate biomarkers of target engagement across immune, vascular, trophic, and network-level domains, alongside example outcome categories that may be most sensitive to restorative effects, and links these mechanistic domains (Table 1) to the endpoint heterogeneity observed across human trials (Tables 3 and 4).

**Table 2:** Mechanism-aligned biomarkers and candidate outcomes for stem cell therapy in TBI. Biomarkers reflect target engagement across immune, vascular, trophic, and network-level repair domains and highlight potential clinical/functional outcomes to consider in mechanism-guided trial design

Mechanistic Target	Candidate Biomarkers Targets	Potential Clinical / Functional Outcomes
Immune Modulation	↓ TNF-α/IL-6; ↑ IL-10 (serum/CSF); microglial shift toward reparative phenotype (↑ M2 markers, ↓ activation markers)	↑ global function scores; ↓ neuropsychiatric symptom burden; attenuated secondary injury trajectory
BBB / Vascular Stabilization	↓ albumin/Evans Blue extravasation; ↑ tight junction proteins (ZO-1/claudin-5/occludin); ↓ MMP-9 / endothelial injury markers	↓ edema/imaging instability; ↓ lesion expansion/tissue loss; faster early neurologic stabilization
Trophic Support	↑ BDNF/GDNF/VEGF/IGF-1 (serum/CSF/tissue); ↓ apoptosis markers (caspase-3/TUNEL); preserved axonal integrity (↑ MBP; ↓ APP)	↑ cognitive performance (domain-specific); ↑ motor recovery (when relevant); ↓ neuronal loss/↑ recovery slope
Network Preservation	DTI integrity (↑ FA, ↓ MD); improved functional connectivity (fMRI coherence); EEG normalization (complexity/connectivity indices)	↑ attention/processing speed/executive control; ↑ functional independence; more durable recovery beyond acute stabilization

In a clinically oriented study using human neural stem cells (hNSCs) in a perinatal/neonatal hypoxic-ischemic injury model, Hartman and colleagues [68] developed an MRI-based algorithm incorporating hierarchical region splitting to noninvasively identify lesion patterns associated with treatment responsiveness. Specifically, the presence of a penumbral region, tissue surrounding the core lesion with preserved structural integrity and molecular “salvageability”, served as a biomarker of an active niche capable of supporting neuroprotective and neurorestorative effects of hNSC transplantation [68]. In contrast, transplantation did not substantially alter the necrotic core, consistent with the concept that tissue regions characterized by rapid and irreversible cell death lack the molecular and structural substrate necessary for functional recovery [68]. These findings support a framework in which treatment response depends not only on the stem cell product itself, but also on the presence of viable perilesional circuitry and neurovascular integrity that can be stabilized and supported by cell-based interventions.

Importantly, stem cell transplantation does not appear to rely solely on long-term engraftment or direct cellular replacement. Rather, therapeutic effects are increasingly understood to arise through paracrine signaling and multi-domain modulation of secondary injury mechanisms, including attenuation of inflammation, suppression of apoptosis, restoration of metabolic homeostasis, stabilization of the BBB, and support of endogenous repair processes [68]. This framework implies that both lesion composition and lesion geometry (e.g., a favorable penumbra-to-core ratio) may influence whether transplanted cells can meaningfully interact with host tissue and support functional recovery [68].

### Stem Cell Migration and Targeting to Injured Tissue

Although the mechanisms governing stem cell migration and targeting remain incompletely characterized, the therapeutic significance of stem cell migration is currently understood within a paracrine-dominant framework [69]. Emerging cell-free approaches, including extracellular vesicle-based therapies derived from MSCs and NSCs, have demonstrated neuroprotective and functional effects mediated through the transfer of growth factors, cytokines, and microRNAs [70]. These findings further challenge the necessity of physical migration to the lesion site as a prerequisite for therapeutic benefit. Nevertheless, perilesional localization likely remains functionally relevant insofar as it may concentrate paracrine signaling, enhance cellular retention within zones of active secondary injury, and position transplanted cells to respond to evolving microenvironmental cues.

Within this context, preclinical work indicates that migratory behavior depends on bidirectional interactions between transplanted cells and host tissue, including injury-associated signals that recruit cells toward the lesion environment [71]. Proposed mechanisms include gradients of chemoattractant factors released from injured tissue and inflammatory microenvironments, which provide directional signaling to transplanted cells [72]. Cytokine signaling has also been implicated as a modulator of migration and proliferation, potentially enhancing recruitment and retention within regions of active secondary injury [72].

Biophysical factors such as galvanotaxis may provide an additional directional mechanism, with stem cells shown to migrate toward the cathode under electrical stimulation and some evidence of increased proliferation [73]. Although additional work is needed to determine whether endogenous post-injury electrical gradients meaningfully guide migration in vivo, galvanotaxis provides a plausible complementary mechanism through which stem cells may exhibit directed motility in injured neural environments [73].

Taken together, cell migration is currently understood not as an independent determinant of efficacy, but as a factor that may modulate the spatial delivery of paracrine mediators within a receptive injury microenvironment. Biomarker-guided stratification, particularly using imaging markers of penumbral integrity, may therefore be essential for optimizing patient selection and maximizing clinical benefit [68].

### Clinical Trials of Stem Cell Transplantation for TBI Treatment

Human trials of cell-based therapy for TBI have largely focused on safety/feasibility, with increasing attention to structural preservation, inflammation-related biomarkers, and domain-specific functional outcomes. The clinical landscape is heterogeneous with respect to injury severity (acute severe vs. chronic deficits), cell product (autologous bone marrow mononuclear cells vs. mesenchymal stromal/stem cells and derivatives), route (intravenous, intrathecal/intranasal, or intracranial), and timing (hours-days vs. months-years post-injury). These design features are not incidental; each implicitly targets distinct mechanisms (e.g., systemic immunomodulation/vascular stabilization in acute TBI vs. circuit-level support and plasticity in chronic focal deficits) [25].

### Literature Search Strategy

To ensure the clinical trials section was current and comprehensive, we performed structured searches in PubMed and ClinicalTrials.gov focused on human interventional studies of stem cell-based therapies for TBI. In PubMed, we combined controlled vocabulary and free-text terms for TBI with cell therapy terms (e.g., stem cell/cell transplantation, mesenchymal stromal cells, bone marrow mononuclear cells, SB623) and trial identifiers (e.g., clinical trial, randomized, sham, placebo, phase), while excluding review articles to prioritize primary clinical reports. In parallel, ClinicalTrials.gov was searched using the condition “Traumatic Brain Injury” with terms capturing major cell products and delivery platforms (e.g., mesenchymal stromal cells, bone marrow mononuclear cells, umbilical cord/Wharton’s jelly, SB623, exosomes/extracellular vesicles), restricted to interventional Phase 1–3 studies with start dates from January 1, 2010 through January 21, 2026. Because indexing and registry terminology can vary across databases, we supplemented the primary searches with targeted route/product-specific queries and citation-chaining from relevant trials, protocols, and high-yield reviews. Using this approach, we identified a focused set of completed and ongoing trials spanning acute and chronic injury stages that illustrate how timing, mechanism, route of delivery, and endpoint selection shape the current clinical landscape of cell-based therapies for TBI. Structured searches were conducted to enhance transparency and reproducibility; however, this article represents a narrative, translational synthesis rather than a formal systematic review or meta-analysis.

### Acute Severe TBI: Intravenous Autologous Bone Marrow Mononuclear Cells

The foundational pediatric trial (“Safety of Autologous Stem Cell Treatment for Traumatic Brain Injury in Children,” NCT00254722) tested early harvest and reinfusion of autologous bone marrow-derived cells in children with severe TBI. The published phase I experience reported that harvest/infusion was feasible and not associated with major infusion-related toxicities, supporting continued development in pediatric acute severe TBI [74].

Subsequent pediatric work has moved beyond feasibility into controlled designs and tissue-level endpoints. In matched-control and randomized/sham-controlled contexts, early bone marrow mononuclear cell (BMMNC) infusion (within ~ 48 h) has been associated with signals in the direction of reduced therapeutic intensity for ICP management, shorter ICU course, and white matter structural preservation/tract connectivity metrics, though interpretation depends on design, dose, and endpoint selection [75, 76].

In adults with severe TBI, a phase I dose-escalation study (NCT01575470) evaluated autologous BMMNC infusion with escalating doses and multimodal outcomes (DTI/structural MRI, inflammatory cytokines, functional measures). The published report supports the feasibility and safety of harvest/processing/infusion and reported group-level signals consistent with tissue preservation and attenuation of pro-inflammatory responses, motivating continued randomized testing [77].

#### Chronic TBI: Intrathecal, Intracranial, and Systemic Approaches

An open-label, non-randomized study of 50 chronic TBI patients evaluated intrathecal autologous BMMNC transplantation combined with individualized neurorehabilitation. At a mean follow-up duration of 22 months, most participants showed symptomatic improvement, with significant pre-post gains on the Functional Independence Measure (FIM). A subset with repeat PET-CT (*n* = 10) demonstrated increased regional brain metabolism broadly, consistent with clinical change. Outcomes appeared more favorable in younger patients, milder injuries, and shorter time since injury, with few serious adverse events. However, efficacy interpretation is limited by the lack of randomization/control and concurrent rehabilitation [78].

One of the most rigorous clinical datasets in chronic TBI comes from the randomized, double-blind, surgical sham-controlled Phase 2 STEMTRA trial of SB623, a modified allogeneic bone marrow-derived MSC product (NCT02416492). In adults with stable chronic motor deficits at least 12 months post-TBI, intracranial implantation of SB623 was well tolerated and met its prespecified primary efficacy endpoint, with significantly greater improvement from baseline on the Fugl-Meyer Motor Scale (FMMS) score at 24 weeks compared to sham controls and maintained function through 48 weeks, without new safety signals [79, 80]. More recently, a post-hoc STEMTRA analysis suggested that optimal SB623 implantation location may vary by lesion substrate, with trends indicating closer-to-lesion placement benefiting motor cortex lesions and more offset placement potentially benefiting deep white matter lesions, highlighting that targeting strategy may be an efficacy modifier in intracerebral cell delivery [81]. This trial provides evidence that SB623 cell therapy can produce meaningful improvements in motor function in chronic TBI, illustrating that focal cell delivery may modulate long-term neurologic deficits even years after injury.

A completed Phase I/IIa trial evaluated repeated intravenous infusions of autologous adipose-derived mesenchymal stromal/stem cells (HB-adMSCs) in adults with chronic TBI (NCT04063215). Registry-reported results support feasibility and tolerability, with generally stable vital signs across infusion visits and follow-up. Exploratory secondary outcomes included functional scales and inflammatory biomarkers, with mixed-model analyses suggesting longitudinal change on the Disability Rating Scale and select cytokines (e.g., IL-4, IFN-γ), although interpretation remains limited by the single-arm design and absence of a peer-reviewed primary outcomes manuscript [82]. Completed trials are summarized in Table 3.

**Table 3:** Completed clinical trials of stem cell-based therapies in TBI. Trials are grouped across injury population and stage, cell product, delivery route, and study design, with headline endpoints summarized

Study (Year)	Population / Stage	Cell product	Route	Design	Key endpoints (headline)
[74] (NCT00254722)	Pediatric severe acute TBI	Autologous BMMNCs	Intravenous	Phase I feasibility/safety	Feasibility, infusion safety, early clinical signals
[75]	Pediatric severe acute TBI	Autologous BMMNCs	Intravenous	Controlled clinical comparison	Therapeutic intensity / intracranial pressure (ICP)-related care burden
[77] (NCT01575470)	Adult severe acute TBI	Autologous BMMNCs	Intravenous	Phase I dose-escalation	Safety, feasibility, inflammatory biomarkers, neuroimaging/functional measures
[76]	Pediatric severe acute TBI	Autologous BMMNCs	Intravenous	Randomized controlled trial	White matter structural preservation (diffusion imaging), ICU course/therapeutic intensity, functional outcomes
[79, 80] (NCT02416492)	Adult chronic TBI with stable motor deficits	SB623	Intracranial (stereotactic implantation)	Phase II randomized, double-blind, sham-controlled	Primary motor endpoint: Fugl-Meyer Motor Scale improvement at 24 weeks; durability through follow-up
[78]	Chronic TBI	Autologous BMMNCs + neurorehabilitation	Intrathecal	Open-label, non-randomized	Functional Independence Measure (FIM), PET metabolic change (subset), safety
[82] (NCT04063215)	Adult chronic TBI	Autologous adipose-derived MSCs	IV (repeated infusions)	Phase I/IIa, single-arm	Safety/tolerability; exploratory functional + cytokines

### Other Clinical sStudies and Registries

Several additional trials and ethics-committee-approved studies have explored alternative stem cell sources (e.g., umbilical cord-derived MSCs), delivery routes (particularly intrathecal administration), and clinically complex populations such as individuals in vegetative states or prolonged disorders of consciousness. Collectively, these investigations support the general feasibility of cell-based approaches and suggest potential functional benefit; however, they vary substantially in control conditions, sample size, and endpoint rigor [80]. As such, they are best interpreted as contributing to safety profiling and hypothesis generation rather than providing definitive evidence of efficacy. Examples include small cohorts using combined local and intravenous MSC delivery with short follow-up periods emphasizing tolerability, randomized designs evaluating intrathecal UC-MSC administration that report functional gains, and larger non-randomized lumbar puncture protocols in severe TBI sequelae that show age-related response patterns, signals that warrant cautious interpretation given the absence of robust controls [25].

#### Trials in Progress and “Mixed Modality” Protocols

Additional stem cell therapy trials for TBI are currently ongoing, with peer-reviewed outcome data pending. A follow-up adult trial (NCT02525432) is designed to test efficacy with placebo/sham controls and richer imaging/biomarker endpoints, including white matter microstructure and longitudinal neurocognitive outcomes [83]. Its design reflects the field’s shift toward linking biologic mechanisms (immune response, neuroinflammation, tissue preservation) to clinically meaningful trajectories. Another ongoing randomized, double-blind, placebo-controlled Phase 2a study of three infusions of HB-adMSCs (NCT05951777) is investigating safety and potential functional and neuroimaging outcomes in chronic TBI [84]. Another Phase 2 trial is recruiting to assess the effects of allogeneic MSCs derived from Wharton’s jelly on systemic immunomodulation and neuroinflammation after TBI (NCT06146062) [85]. In a complementary approach, the MATRIx trial is a phase 2 dose-finding study evaluating the safety and efficacy of intravenous allogenic BM-MSCs administered within 48 h of severe TBI, with dose-finding across two cell concentrations (NCT06163833) [86]. These ongoing studies emphasize feasibility and mechanistic endpoint selection rather than definitive efficacy at this time. Ongoing clinical trials are summarized in Table 4.

**Table 4:** Ongoing clinical trials of cell-based therapies for TBI. Summary of active registered studies by population/stage, cell product, delivery route, design, and primary endpoints

Trial (NCT)	Population / Stage	Cell product	Route	Design	Primary endpoint(s)
[84] Autologous Bone Marrow Mononuclear Cells for Adult Severe TBI (NCT02525432)	Adults, severe acute TBI	Autologous BMMNCs	Intravenous	Multicenter, randomized, blinded, placebo-controlled (sham harvest)	MRI/DTI structural preservation (gray/white matter volume + integrity)
[86] TRAUMACELL: Wharton’s Jelly UC-MSCs in Severe TBI (NCT06146062)	Acute severe isolated TBI	Allogeneic Wharton’s jelly-derived UC-MSCs	Intravenous (repeated infusions)	Randomized, double-blind, placebo-controlled	Neuroinflammation (Translocator Protein PET-MRI)
[85] Hope Biosciences HB-adMSC in Chronic TBI (Phase 2a) (NCT05951777)	Chronic TBI	HB-adMSCs	Intravenous (3 infusions)	Randomized, double-blind, placebo-controlled	Safety + functional outcomes
[87] MATRIx: Allogeneic BM-MSCs in Acute Severe TBI (NCT06163833)	Adults, acute severe TBI	Allogeneic BM-derived MSCs	Intravenous (single infusion within 48 h)	Multicenter, randomized, double-blind, placebo-controlled	Safety + efficacy (dose optimization)

Across both acute and chronic TBI populations, the most consistent finding from human studies is that cell-based interventions, particularly autologous bone marrow mononuclear cell infusions in acute severe TBI and intracranial delivery of MSC-derived products in chronic focal deficits, are feasible and generally well tolerated when implemented under controlled clinical protocols. Across several designs, these approaches have been associated with converging signals of structural preservation and selected functional improvements, although the magnitude and consistency of benefit vary across studies and outcome domains [76, 77, 80].

At the same time, the literature remains constrained by heterogeneity in injury phenotype, timing of administration, dose, route, and endpoint selection, as well as the frequent use of open-label designs or multimodal protocols that limit causal attribution to the cellular intervention itself [25, 87]. Rather than diminishing the translational rationale, these limitations reinforce the central premise of this review: meaningful clinical efficacy will likely depend on improved patient stratification, biomarker-guided targeting, and mechanism-aligned outcome measures, rather than expecting uniform effects across the full spectrum of TBI presentations.

## Conclusions

TBI is a dynamic disorder driven by secondary cascades, including neuroinflammation, BBB disruption, oxidative stress, apoptosis, and network-level dysfunction, that unfold over time and shape long-term outcomes. Yet, many clinical efforts have pursued single-target interventions, contributing to translational difficulty and a persistent lack of consistently effective therapies [4]. Progress in neurorestorative treatment will likely require approaches that support coordinated, system-level repair across interacting biological domains. In this context, stem cell-based therapies represent a promising alternative. Converging evidence suggests their effects are mediated primarily through paracrine restorative signaling that promotes immunomodulation, trophic support, angiogenesis/neurovascular stabilization, and synaptic plasticity, rather than direct cellular replacement [25]. Stem cells may therefore be best conceptualized as context-sensitive neurobiological response modifiers. Critically, functional benefit appears to depend on host receptivity, including tissue viability, penumbral integrity, immune milieu, and vascular state, at least as much as on cell product potency [68].

Across the human literature, cell harvesting, processing, and delivery have been generally feasible and well tolerated under controlled protocols [74, 77]. Signals of efficacy are present, but they are not uniform and appear contingent on injury phenotype, timing, route of administration, and the outcome domains prioritized. In acute severe TBI, the most consistent signals to date tend to involve structural preservation and biological engagement (e.g., white matter integrity, inflammatory markers) rather than large and immediate clinical effect sizes [75, 76]. In chronic TBI, the strongest evidence currently supports durable motor improvements when delivery is targeted [79]. Importantly, mixed results across studies should not be dismissed as noise; they likely reflect meaningful differences in injury stage, the availability of salvageable tissue, dosing strategy, route, and endpoint selection. This interpretation is consistent with aggregate preclinical evidence across multiple independent meta-analyses, which collectively confirm that delivery timing and route are among the strongest mediators of stem cell efficacy in experimental TBI models [37–39]. As such, these variables must be treated as mechanistic design features rather than secondary details [87].

The translational framework presented in this review addresses this complexity by explicitly aligning four interdependent dimensions: injury stage, biological target, route of administration, and outcome selection. When applied, stem cell therapy becomes a useful model system for testing when the injured brain is receptive to restorative intervention and for clarifying how to match therapy to dominant biology at a given phase of recovery. This perspective implies that the next gains in the field will come less from “one-size-fits-all” trials and more from mechanism-aligned patient stratification and target engagement.

Future work will likely benefit from biomarker-gated trials in which enrollment requires evidence of a receptive substrate [68]. Promising stratifiers include imaging markers (e.g., penumbra-to-core features, diffusion-based indices of white matter integrity), inflammatory/immune signatures (e.g., cytokine profiles), and clinically meaningful phenotypes (e.g., stable focal deficits vs. diffuse multi-domain impairment; time since injury; age; injury severity). Notably, at chronic timepoints, route of administration appears to be a critical determinant of target engagement [40, 42, 43]. In practice, matching therapy to salvageable tissue and networks may be the difference between detecting a reliable signal and averaging it away.

Several limitations of the present literature should be mentioned. Many studies remain small, heterogeneous, and, in some cases, uncontrolled, which constrains causal inference. Multimodal protocols and variability in endpoints further complicate synthesis and may obscure true target engagement. Many studies may be limited not only by statistical power, but by the challenge of selecting outcomes that are sensitive to the mechanisms most likely engaged by a given cell product, delivery route, and treatment window. It should also be acknowledged that some candidate stratifiers discussed here, such as BBB permeability measures, rely on methods or biosamples not readily accessible in clinical or regulatory contexts. Translating this framework into practice will require using validated and feasible surrogate markers obtainable from blood, CSF, or neuroimaging for biomarker-gated enrollment. In addition, biological moderators such as sex, comorbidities, polytrauma, and repeated mTBI are under-modeled and should be incorporated more systematically in future research.

Overall, stem cell therapies have crossed a threshold of clinical plausibility, with an accumulating safety and feasibility record and multiple converging mechanistic signals. The central question is no longer whether stem cells work in a general sense, but rather: for whom, when, by what mechanisms, and with what delivery strategy can cell-based interventions reliably produce meaningful recovery in TBI. Answering these questions will require trials that move beyond broad efficacy testing toward biologically informed patient stratification, mechanism-aligned delivery strategies, and outcome measures capable of capturing target engagement across recovery stages.

## Data Availability

No datasets were generated or analysed during the current study.
